# Proteoglycans involved in bidirectional communication between mast cells and hippocampal neurons

**DOI:** 10.1186/s12974-019-1504-6

**Published:** 2019-05-20

**Authors:** Juan Antonio Flores, María Pilar Ramírez-Ponce, María Ángeles Montes, Santiago Balseiro-Gómez, Jorge Acosta, Guillermo Álvarez de Toledo, Eva Alés

**Affiliations:** 1 Dpto. de Fisiología Médica y Biofísica, Facultad de Medicina, Universidad de Sevilla, Av. Sánchez Pizjuán 4, 41009 Sevilla, Spain; 2 Present Address: Department of Neuroscience, Yale School of Medicine, 295 Congress Avenue, New Haven, CT 06510 USA

**Keywords:** Ca^2+^ imaging, neuro-immune, hippocampal neurotransmission, NMDA receptors, proteoglycans, exocytosis

## Abstract

**Background:**

Mast cells (MCs) in the brain can respond to environmental cues and relay signals to neurons that may directly influence neuronal electrical activity, calcium signaling, and neurotransmission. MCs also express receptors for neurotransmitters and consequently can be activated by them. Here, we developed a coculture model of peritoneal MCs, incubated together with dissociated hippocampal neurons for the study of cellular mechanisms involved in the mast cell-neuron interactions.

**Methods:**

Calcium imaging was used to simultaneously record changes in intracellular calcium [Ca^2+^]_i_ in neurons and MCs. To provide insight into the contribution of MCs on neurotransmitter release in rat hippocampal neurons, we used analysis of FM dye release, evoked by a cocktail of mediators from MCs stimulated by heat.

**Results:**

Bidirectional communication is set up between MCs and hippocampal neurons. Neuronal depolarization caused intracellular calcium [Ca^2+^]_i_ oscillations in MCs that produced a quick response in neurons. Furthermore, activation of MCs with antigen or the secretagogue compound 48/80 also resulted in a neuronal [Ca^2+^]_i_ response. Moreover, local application onto neurons of the MC mediator cocktail elicited Ca^2+^ transients and a synaptic release associated with FM dye destaining. Neuronal response was partially blocked by D-APV, a *N*-methyl-D-aspartate receptor (NMDAR) antagonist, and was inhibited when the cocktail was pre-digested with chondroitinase ABC, which induces enzymatic removal of proteoglycans of chondroitin sulfate (CS).

**Conclusions:**

MC-hippocampal neuron interaction affects neuronal [Ca^2+^]_i_ and exocytosis signaling through a NMDAR-dependent mechanism.

## Background

Communication between the immune and the nervous system is considered an important biological process [1, 2]. While microglia are the resident immune cells in the brain and play a pivotal role in immune surveillance in the central nervous system (CNS) [3, 4], the roles of other immune cells remain unexplored. Mast cells (MCs) are also resident in the brain of all mammalian species studied [5] and located in the meninges and perivascular area on the brain side of the blood-brain barrier [6] and the parenchyma of the thalamic and hippocampal regions [7, 8]. A key feature of MCs is that they contain in their cytoplasm numerous secretory granules that hold a vast array of inflammatory mediators and biologically active substances such as histamine, serotonin, proteoglycans, and proteases. When appropriately activated, MCs undergo degranulation, a process by which the preformed granule compounds are rapidly released into their surroundings [9]. MC degranulation can occur in response to various external stimuli including IgE receptor crosslinking, complement activation, neuropeptides, and certain toxins [10]. MCs are also temperature and pressure sensitive, degranulating in response to noxious physical stimuli through a mechanism dependent on transient-receptor-potential channel 2 [11]. In addition to inducing the release of preformed granule constituents, MC activation leads to de novo synthesis of many bioactive compounds, including lipid mediators such as leukotrienes or prostaglandins or a variety of cytokines, chemokines, and growth factors [10, 12]. Overall, preformed granular material is released in minutes, while de novo materials are secreted within hours*.* Notably, MC activation does not necessarily lead to degranulation. For example, exposure of MCs to lipopolysaccharide can cause the release of cytokines without observable degranulation [13].

The mast cell-nerve relationship has been discussed extensively [14–16]. MCs are often found in close proximity to nerves [17]. When stimulated, messenger molecules released from nerves (e.g., neuropeptides) can elicit MC degranulation, but also mast cell-derived mediators (e.g., histamine, serotonin) can modulate neurotransmission [18], creating a bidirectional positive feedback-loop that may result in neurogenic inflammation [19]. MCs and their mediators are important conveyors of the communication between the innate enteric immune system and the enteric nervous system, and there is strong evidence for nerve-MC interaction, particularly in gastrointestinal diseases such as inflammatory bowel syndrome [20, 21].

The functions of MCs in the brain are unknown. However, clear evidence indicates brain MCs are important contributors to neuropsychiatric states such as anxiety [22] and are able to modulate neuroinflammation, which may affect diseases such as multiple sclerosis and Alzheimer’s disease [2, 23]. The interaction between MCs and CNS neurons has been little studied. We examined the possibility of a bidirectional communication between hippocampal neurons and MCs through the use of a coculture model, where rat peritoneal mast cells, representative of connective tissue mast cells [24], are incubated with dissociated hippocampal neurons. A similar model was developed for the study of the cellular mechanisms involved in the associations between MCs and nerves in vivo [17] and MCs and sympathetic neurons [25, 26]. Hippocampal neurons were activated by depolarization and MCs were immunologically activated (by antigen) as well as by the compound 48/80 and LPS. After selective stimulation of MCs and neurons, we used [Ca^2+^]_i_ imaging to record subsequent cell activation. Besides, a cocktail of MC secretory products was applied directly onto neuronal somata causing [Ca^2+^]_i_ transients and exocytosis. This response was NMDAR-dependent. We show for the first time bidirectional communication between hippocampal neurons and MCs and a role of MCs in neurotransmission.

## Methods

### Preparation of neuronal cultures

Hippocampal neurons were cultured from brains of P0–P1 newborn rats isolated by surgical separation of the cranial bones by natural suture. CA-1 and CA-3 hippocampal regions from both sides were isolated under a light surgical microscope and removed from the brain. The two hippocampi were immersed in 1 ml of enzymatic solution and kept under continuous agitation (80 rpm) for 40 min and centrifuged at 200×*g* for 5 min. The enzymatic solution contained 1 ml of DMEM (PAA Laboratories), 0.2 mg of cysteine, 1 mM CaCl_2_, 0.5 mM EDTA and 5 units/ml papain (Worthington). The enzymatic solution was previously bubbled with 95% O_2_ and 5% CO_2_ for 20 min. After centrifugation, the supernatant was removed, and 1 ml of inactivating solution was added to the tissue to prevent further digestion. The inactivating solution contained 800 μl of DMEM (Gibco), 200 μl FBS (BioWest), 200 units of penicillin (Gibco), 200 μg of streptomycin (Gibco), 2.5 mg albumin and 2.5 mg trypsin inhibitor (Sigma). After this process, the tube was centrifuged again at 200×*g* for 5 min and mechanically dispersed afterward with a 1-ml pipette tip five times. The supernatant was discarded and replaced with neuronal media, containing: 1 ml Neurobasal (Gibco), 20 μl B27 (Gibco) (50×), 10 μl Glutamax (Gibco) (100×), 20 units penicillin and 20 μg streptomycin. Neurons were plated at a density of 150,000 cells/well (12-well plates) over a single feeding layer of astrocytes grown on 15 mm *ϕ* coverslips. For the astrocyte feeding layer, the coverslips were previously treated with 1 ml of an acidic solution containing 17 mM acetic acid, 0.8 mg collagen (BD Biosciences), and 1 mg poly-D-lysine (Sigma). For the treatment, the solution was gently spread over the coverslips with a cotton bud and let to dry overnight and washed with water three times. The feeding layer was made of astrocytes from rat and seeded on the cover slips at a density of 40,000 cells/dwell for 5 days. The astrocyte feeding layer was preserved by adding uridine solution that prevents cellular division. The uridine solution contained DMEM, 20.4 mM uridine (Sigma) and 8.1 mM 5-phluoro-2-deoxy-uridine (Sigma). Neurons were cultured between 14–17 DIV in an incubator at 37 °C with an atmosphere of 95% O_2_ and 5% CO_2_.

### Mast cell isolation and preparation of cocultures

MCs were isolated from the peritoneal cavity of 3- to 5-month-old rats following a procedure described in detail elsewhere [27]. The peritoneal wash fluid contains a large number of cells, a small fraction of which are MCs. Using a Percoll (Sigma-Aldrich) gradient, fully differentiated connective tissue peritoneal MCs were separated from other cells [28]. Freshly purified peritoneal MCs were added to the neuronal cultures at 3 × 10^4^ cells per well 18–24 h before the experiment. To induce antigen stimulation, 4 h after seeding, peritoneal MC were sensitized with mouse monoclonal anti-DNP IgE (1 μg/ml). All reactives and drugs were purchased from Sigma, unless otherwise stated.

### Mediator cocktail released in response to heat

1 × 10^6^ purified MCs obtained after Percoll purification were sedimented by centrifugation (200×*g*, 5 min) and resuspended in 1 ml of basal Locke solution. The basal Locke solution contained 140 mM NaCl, 10 mM HEPES, 3 mM KOH, 2 mM CaCl_2_, 1 mM MgCl_2_, and 10 mM glucose. The pH of the solution was adjusted to 7.3 with NaOH. Cells were placed into a prewarmed Thermomixer (Eppendorf) for 1 h at 53 °C with gentle agitation. MC degranulation was checked by visual inspection under a microscope. Cells were separated from supernatants by centrifugation at 200×*g* for 5 min. Supernatants were aliquoted (50 μl) and stored at − 80 °C until use.

### Recordings of [Ca^2+^]_i_ signal

Changes in [Ca^2+^]_i_ were monitored by dual excitation microfluorimetry in isolated hippocampal neurons or in cocultured MC-hippocampal neurons incubated in Locke solution containing Fura-2 AM (Molecular Probes) (5 μM for 45 min at 37 °C in the dark) and pluronic F-127 (Sigma) (final concentration 0.004%). Then, cells were washed twice in Locke external solution without the probe and used for imaging. The coverslip with cells loaded with Fura-2 AM was then mounted in a RC-25F perfusion chamber (Warner instruments) and placed on an inverted microscope (AxioVert 200, Zeiss) equipped with a standard filter set (XF04-2; Omega Optical). During recordings, cells were excited by a xenon light source at 360/380-nm wavelengths (exposure time, 0.5 s; data acquisition at 0.33 Hz) by means of two narrow beam band-pass filters selected by a computer-controlled wheel. The emitted fluorescence was filtered through a 520-nm filter, captured with an ORCA-R2 CCD camera (Hamamatsu Photonics). Data were acquired and stored using HCImage software and exported to Igor Pro (WaveMetrics) for analysis. All values were normalized to the basal fluorescence (baseline). The Ca^2+^_i_ signal was expressed by the ratio of fluorescence at 360 nm and 380 nm. All experiments were performed at 37 °C.

### Imaging and analysis of FM dye release

Fifteen-millimeter cover glasses with cultured hippocampal neurons (days in vitro (DIV) 12–16) were mounted in the perfusion chamber. Synaptic vesicles were loaded by substitution of basal Locke solution with 70 mM [K^+^] Locke solution (generated by equimolar replacement of NaCl by KCl) containing 4 μM FM1-43 (Molecular Probes) for 2 min. After washing in a basal Locke external solution for 10 min, synaptic release from selected neurons was induced by applying 70 mM [K^+^] Locke solution or MCM through a pressure pulse (5 s) with a micropipette (1 μm *ϕ*) positioned ~ 100 μm to the neuronal soma. Images were obtained using a 63× PlanNeofluar (NA 1.3) oil immersion lens (Zeiss) on an Axiovert 200 inverted microscope equipped with a standard filter set for FM1-43 dye (XF115-2; Omega Optical). Images were captured with an ORCA-R2 CCD camera (Hamamatsu Photonics) controlled by HCImage software (Hamamatsu Photonics). Time-lapse images were acquired at a rate of 0.5 Hz, 1344 × 1024 pixels (binned 1 × 1), with an exposure time of 200 ms. Recordings were performed at 37 °C.

For quantification of FM dye destaining, circular regions of interest (ROI, 5 × 5 pixels) were located manually over the center of FM dye fluorescence of spatially separated synapses. Raw destaining trace values were extracted and loaded into Igor Pro (Wavemetrics) for further analysis. The destaining traces were graphed as the change in fluorescence (*ΔF*, absolute values) normalized to the fluorescence at the start of the experiment (*F*0) using the following equation: *ΔF*/*F*0 = (FF − *F*0)/*F*0, where FF is the point at which the destaining curve slope becomes zero. The FM dye release curves of single synapses were fitted mono-exponentially. A successful mono-exponential fit was defined by positive values for amplitude (*ΔF*) and time constant (*τ*).

### Statistical analysis

Differences between groups were tested for statistical significance using Student’s *t* test for unpaired data when the data passed a normality test (Lilliefors-corrected Kolmogorov–Smirnov) and a Mann–Whitney *U* test when it did not [29]. The data were regarded as statistically different when *P* < 0.05. All statistical analyses were performed using SPSS Statistics 22 software (IBM). All data were plotted as the mean ± SEM. The presented data represent experiments performed with cells from at least three different cultures.

## Results

### Bidirectional communication between hippocampal neurons and MCs in coculture

**Fig. 1:**
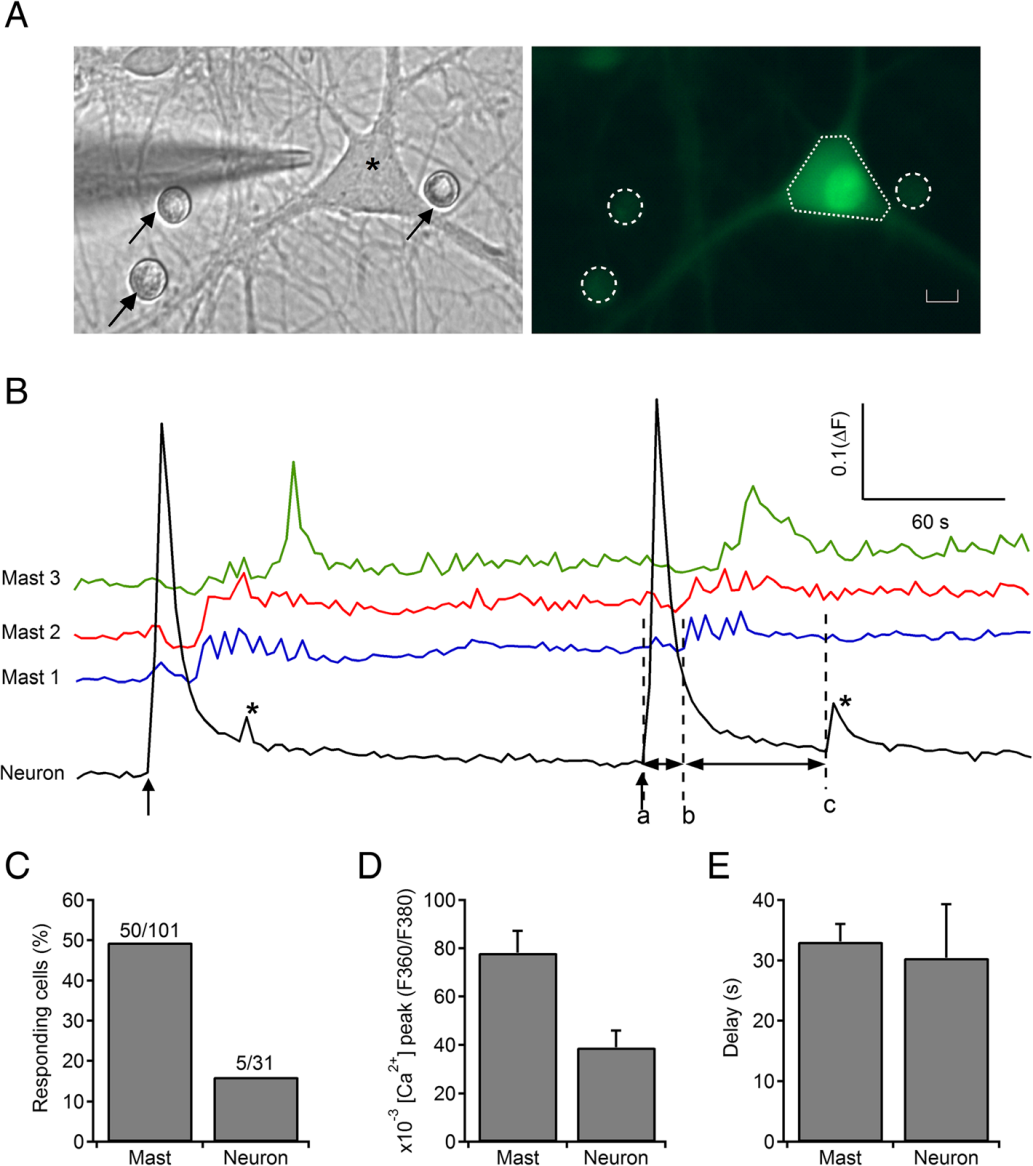
Calcium imaging of MC and Neuronal activity. **a** Phase-contrast image on the left panel of a hippocampal neuron (asterisk) at 2 weeks in culture following 24 h coculture with mast cells (black arrows). A glass micropipette of 1 μm *ϕ* was positioned very close to the neuronal soma before pressure ejection (2.5 psi) pulses of 5 s. The right panel shows the same cells loaded with the Ca^2+^ sensitive dye Fura-2 AM (MCs selected by dashed circles and neuron by the dotted polygon). Scale bar is 5 μm. **b** Traces of [Ca^2+^]_i_ transients in three mast cells (blue, red, and green traces) and a hippocampal neuronal cell body (black trace) triggered by the initial neuronal depolarization by microperfusion (70 mM [K^+^] Locke solution). Note the [Ca^2+^]_i_ oscillation pattern elicited in MCs after initial neuronal activation and the short [Ca^2+^]_i_ transient (*) evoked in neuron later. Both responses were reproduced in a second stimulation. **c** Percentage of responding cells. **d** Mean [Ca^2+^]_i_ peak obtained in MC- and neuron-evoked responses. **e** Latencies measured from the beginning of neuronal [Ca^2+^]_i_ transient induced by depolarization (**a**) to the origin of MC oscillations (**b**) and from the latter to the second rise in neuronal trace (**c**) (*)

When in a coculture of mixed hippocampal neurons and MCs, loaded with FURA-2 AM, we stimulated a neuron by direct depolarizing stimuli applying a pressure pulse of K^+^-enriched solution (70 mM KCl with iso-osmotic reduction of NaCl) (Fig. 1a), a high [Ca^2+^]_i_ transient was evoked upon the onset of the stimulus in neuronal cell body, as it was expected (Fig. 1b). The peak amplitude (*Δ*ratio of F360/F380) of Ca^2+^ response was of 0.55 ± 0.06 a.u. After a short latency, nearly 50% of surrounding MCs (a number of 50 MCs, out of 101) experimented [Ca^2+^]_i_ oscillations of variable pattern (Fig. 1b) and low amplitude (*ΔF*/*F*, 0.078 ± 0.009) (Fig. 1d). No calcium signal was observed when the depolarizing pulse was applied on a pure MC culture. Interestingly, after MC responses, a new but short [Ca^2+^]_i_ transient was observed in 16% of tested neurons (5 out of 31). This neuronal signal only occurred in neurons cocultured with MCs but not in isolated hippocampal neurons, and it could be reproduced for a second time when a new depolarizing pulse of high K^+^ was again applied, suggesting that MC mediators feedback on neuronal activity. The amplitude of the second neuronal [Ca^2+^]_i_ peak was 0.039 ± 0.007. While MCs responses occurred 33.1 ± 2.9 s after the onset of first neuronal transient induced by depolarization, late Ca^2+^ transients evoked in neurons after MCs response showed a similar delay of 30.4 ± 8.8 s (Fig. 1e).

### Activation of hippocampal neurons by mast cell degranulation

**Fig. 2:**
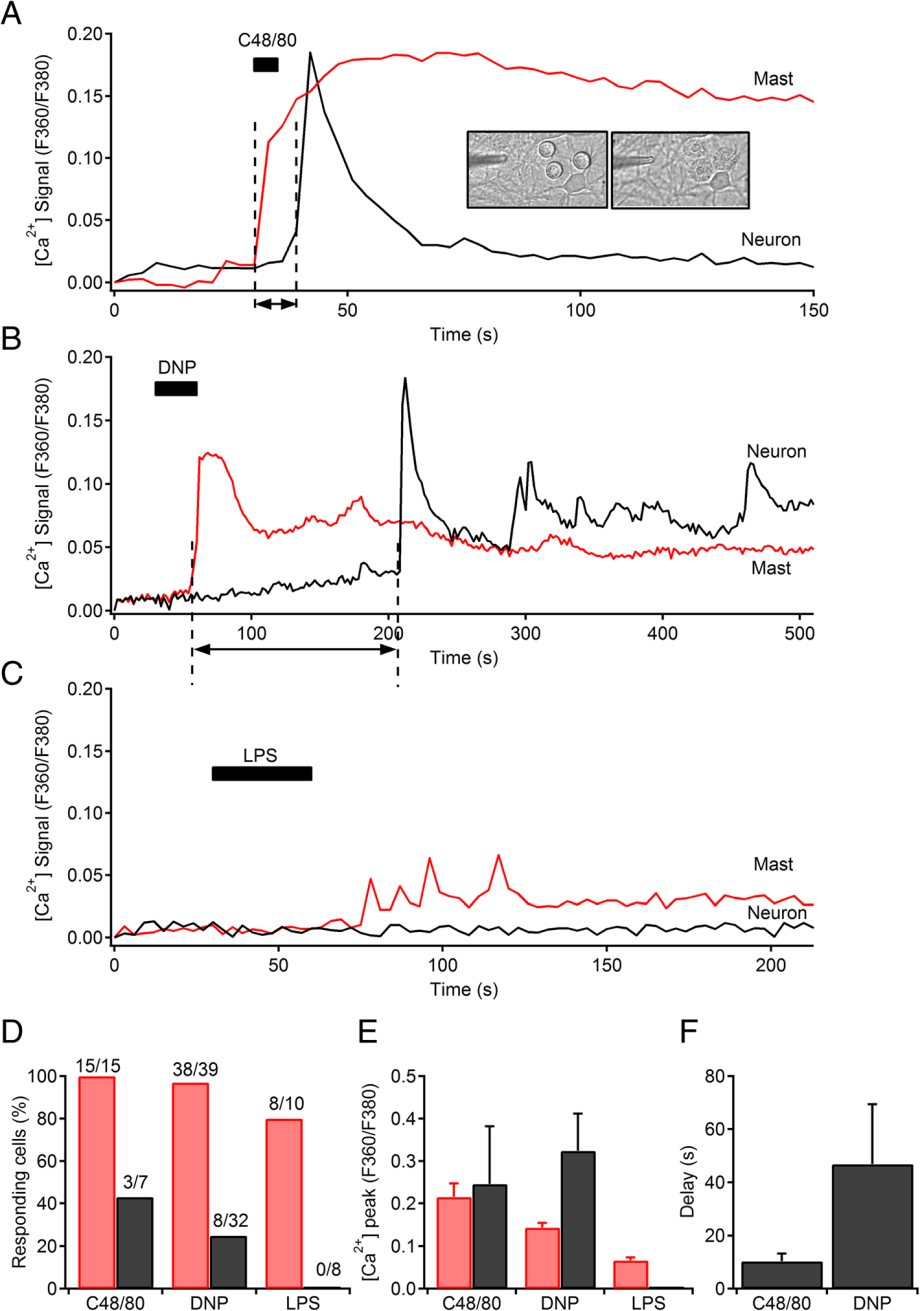
MC to neuronal [Ca^2+^]_i_ signaling. **a** Traces of [Ca^2+^]_i_ transients in a mast cell (red trace) and a hippocampal neuronal cell body (black trace) in response to mast cell activation by C48/80 microperfusion (100 μg/ml for 30 s). Inset shows pictures of MCs morphology and appearance before and after stimulation. **b** Traces of [Ca^2+^]_i_ transients in a mast cell (red trace) and a hippocampal neuron (black trace) in response to mast cell activation by FcεRI receptor crosslinking by DNP microperfusion (1 μg/ml for 30 s). **c** Traces of [Ca^2+^]_i_ transients in a MC (red trace) and a hippocampal neuronal cell body (black trace) in response to MC activation by LPS (1 μg/ml for 1 min). **d** Percentage of responding cells to C48/80, DNP, and LPS. **e** Mean [Ca^2+^]_i_ peak obtained in MCs (red bars) and hippocampal neurons (black bars) evoked by C48/80, DNP, and LPS. **f** Latencies measured by the time interval between the rise of MC [Ca^2+^]_i_ transient and the onset of neuronal [Ca^2+^]_i_ response

**Fig. 3:**
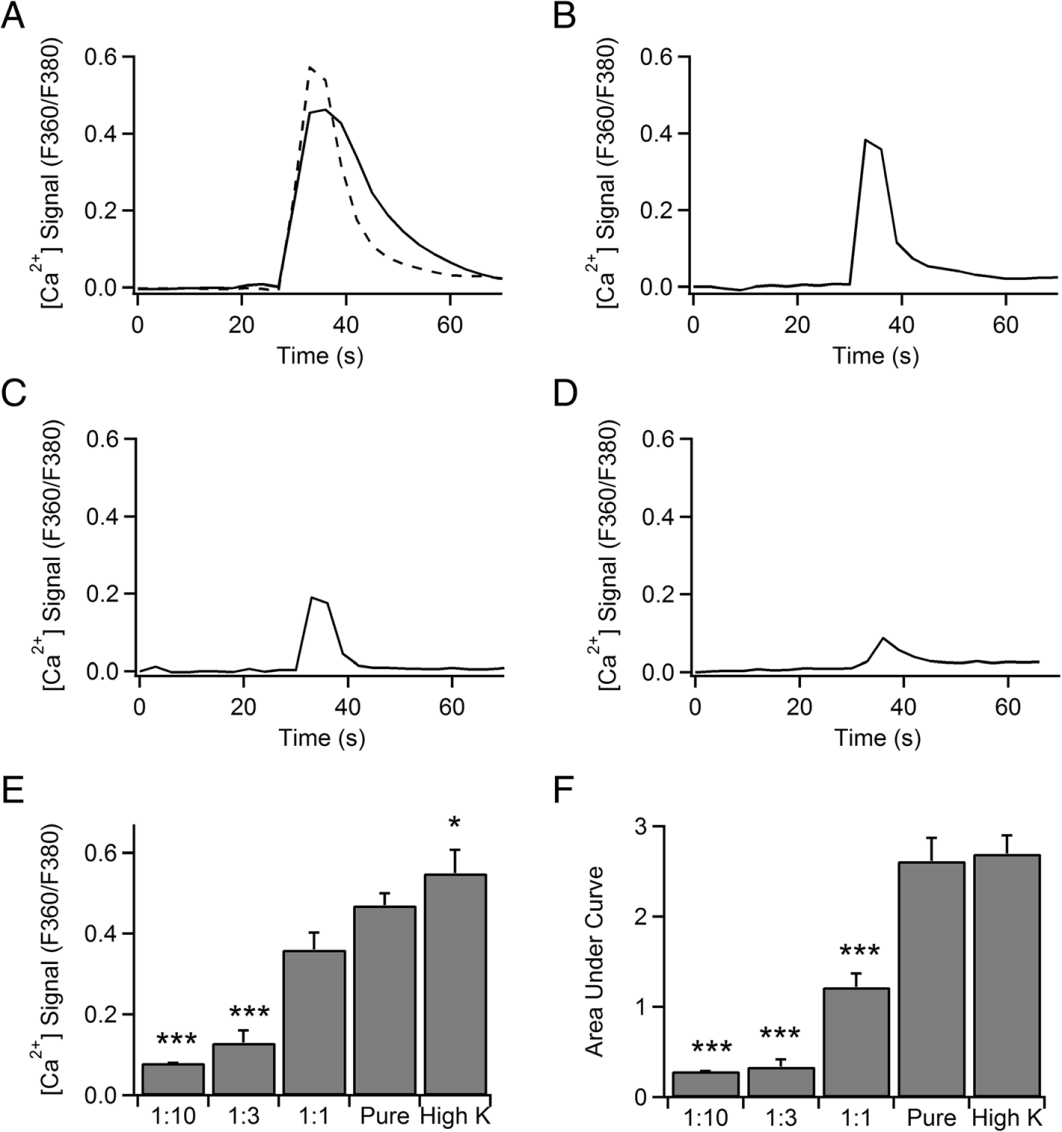
Mast cell mediator cocktail (MCM) directly elicits [Ca^2+^]_i_ transients in hippocampal neurons. **a** [Ca^2+^]_i_ signals evoked by single pressure ejection pulses of 5 s of pure extract of MCM (solid trace) and 70 mM [K^+^] Locke solution (dashed trace). The resulting calcium responses obtained by MCM was compared with calcium responses elicited by depolarization. **b** Representative [Ca^2+^]_i_ signal evoked by a single pressure ejection pulse of 5 s of MCM at 1:1 dilution. In order to estimate the minimal number of MCs necessary to obtain enough amount of mediators to induce a calcium transient in neurons, different dilutions from pure MCM were made. **c** Representative [Ca^2+^]_i_ signal evoked by a single pressure ejection pulse of 5 s of MCM at 1:3 dilution. **d** [Ca^2+^]_i_ signal evoked by a single pressure ejection pulse of 5 s of MCM a 1:10 dilution. **e** Mean [Ca^2+^]_i_ peak obtained in neuronal responses evoked by 70 mM [K^+^] and MCM at 1:0 (pure), 1:1, 1:3, and 1:10 dilutions. **f** Mean area under curve obtained from [Ca^2+^]_i_ transients in neurons evoked by 70 mM [K^+^] and MCM at 1:0 (pure), 1:1, 1:3, and 1:10 dilutions. The latter was the lowest effective dilution capable of stimulating neurons. *p < 0.05, ***p < 0.001

The selective stimulation of MCs in close proximity to hippocampal neurons by compound 48/80 and antigen caused an immediate change in appearance of most MCs that led to degranulation within minutes. When MCs were activated with C48/80 (5 s application), a rapid Ca^2+^ elevation was elicited (*ΔF*/*F*, 0.21 ± 0.03) that persisted at mid-levels for minutes (Fig. 2a). The effect of MC degranulation by C48/80 on Ca^2+^ signals of neighboring neurons occurred fairly rapidly (delay, 10.3 ± 2.9 s) (Fig. 2f) and consisted in an acute Ca^2+^ transient with a peak amplitude of 0.24 ± 0.13 (Fig. 2e). The percentage of responding neurons was totaled 43%. MCs previously sensitized with monoclonal IgE against dinitrophenyl (DNP) and stimulated with the multivalent antigen DNP-conjugated human serum albumin (HSA) showed a mild degranulation response evaluated by visual inspection of images using a bright-field microscopy. Antigenic stimulation (DNP) for 30 s elicited [Ca^2+^]_i_ oscillations in MCs (Fig. 2b) with a peak amplitude of 0.14 ± 0.01. Subsequently, 25% of hippocampal neurons responded after MC activation, since Ca^2+^ waves of variable amplitude (*ΔF*/*F*, 0.32 ± 0.08) (Fig. 2e) and delay (mean, 46.8 ± 22.6 s) (Fig. 2f) were observed. The application of LPS for 1 min to MCs only evoked a short burst of oscillations in [Ca^2+^]_i_ of low amplitude (*ΔF*/*F*, 0.06 ± 0.007) and took a long time to appear (Fig. 2c). MCs did not show morphological changes or apparent degranulation, and [Ca^2+^]_i_ signals from adjacent neurons remained unaltered. The possibility of direct effects of DNP or C48/80 on neuronal [Ca^2+^]_i_ signal was tested by applying these agents to hippocampal cultures in the absence of MCs. Neither DNP nor C48/80 alone had an effect on neuronal [Ca^2+^]_i_ signals. However, C48/80 has been said to exert a direct and strong excitatory action on enteric neurons and visceral afferents [30]. Therefore, in preventing potential direct effects of these agents on neurons, or indirectly, via mixed glia, we prepared a cocktail of secretory mediators from MCs (MCM). The cocktail of MC mediators (MCM) consisted of an extract obtained from degranulation by heat (53 °C, for 1 h) of 10^6^ MCs per milliliter. Application of this MCM via a local pipette directly onto a hippocampal neuron cell body evoked a successful activation, measured by [Ca^2+^]_i_ transients, in 92% of cells (*n* = 121) (Fig. 3a). Hippocampal neurons from a pure culture without glia were also able to react to the MCM similarly to neurons from mixed cultures. As a control, when the cocktail of mediators was obtained from MCs without degranulation (subjected to 37 °C instead of 53 °C, for 1 h), it failed to induce neuronal activation (*n* = 10). Therefore, hereinafter, we used the MCM obtained by heat (53 °C). In comparison with pure MCM, application of high K^+^-enriched solution evoked higher [Ca^2+^]_i_ transients with larger peaks (*ΔF*/*F*, 0.55 ± 0.06 vs 0.47 ± 0.03; *p* = 0.021) (Fig. 3e) but a similar area under curve (or the integral over the deviation in [Ca^2+^] throughout the transient) (2.62 ± 0.25 vs 2.7 ± 0.2 ) (Fig. 3f). Successive dilutions of this MCM concentrate (1:1; 1:3; 1:10) showed a dose response-dependence: [Ca^2+^]_i_ peak amplitude decreased from 0.36 ± 0.04, *n* = 18 cells; (1:1 dilution) to 0.13 ± 0.03, *n* = 16 cells; (1:3 dilution) whereas the area under the curve decreased from 1.22 ± 0.15 (1:1 dilution) to 0.34 ± 0.08 (1:3 dilution). The last dilution of MCM (1:10) evoked a response in only 1 out of 9 tested neurons that showed a [Ca^2+^]_i_ peak amplitude of 0.8 and an area under the curve of 0.29.

### MC mediators elicit synaptic transmission in hippocampal neurons

**Fig. 4:**
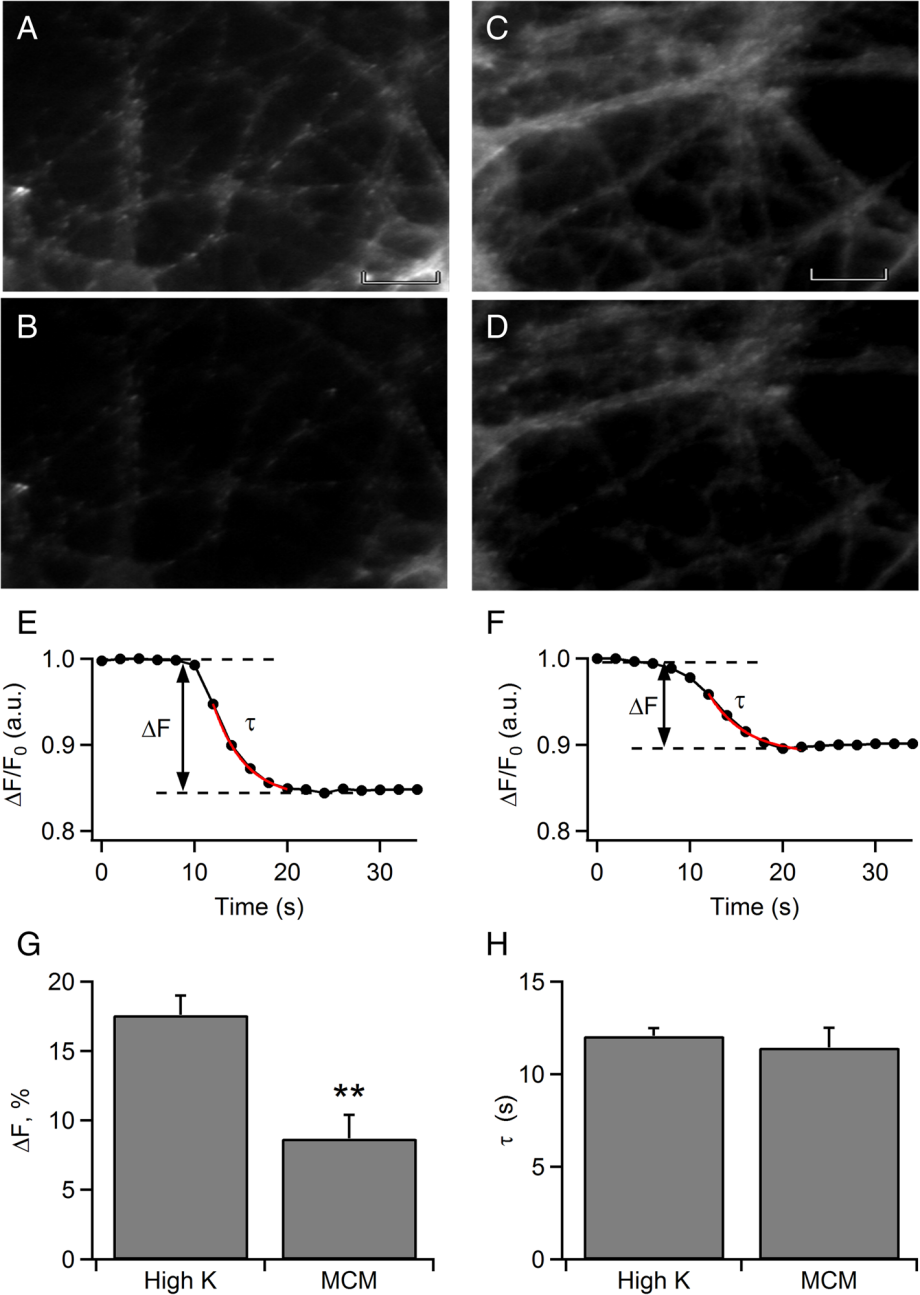
Results of FM imaging system in cultured hippocampal neurons. **a**, **c** Synaptic boutons of hippocampal neurons stained by FM1-43 dye in a loading process which consisted in 2 min of stimulation with 70 mM [K^+^ ] and subsequent washing. The presence of numerous puncta of fluorescence that appear to be lined up along the neurites result from localized internalization of the dye and are not washed out. **b** Synaptic boutons destained after a stimulation pulse of 5 s with 70 mM [K^+^]-enriched solution or **d** with MCM. Note the marked decrease in fluorescence intensity of most of the puncta visible in **a** and **c**. Scale bar is 5 μm and applies to all frames **e** Normalized destaining curves of FM dye loaded synapses upon stimulation with high K^+^ and **f** MCM. FM dye release curves of all single synapses were fitted mono-exponentially. **g** Amplitude (*ΔF*) and **h** *τ* were measured from monoexponential fitting curves obtained by depolarization and MCM, and plotted. Data represent average values of 6 (70 mM [K^+^ ]) and 14 (MCM) neurons. **p < 0.01

Transmitter release is tightly regulated by the presynaptic Ca^2+^ transient [31, 32]. Because MC degranulation is capable of evoking evident Ca^2+^ transients in hippocampal neurons, we examined the transmitter of release using fluorescent imaging. Activity-dependent unloading of fluorescent styryl dyes (FM dyes) from recycling synaptic vesicles provides a direct measure of synaptic function at the level of single synapses [33]. Synaptic vesicles were loaded with FM1-43 dye for 60-s bath perfusion using 70 mM [K^+^] solution. Subsequently, cells were continuously perfused for 5–10 min with 3 mM [K^+^] basal solution in order to wash out excess FM dye from neuronal membranes. The cells showed a substantial amount of internalized probe (Fig. 4a, c) as a consequence of a first exo-endocytic process. Synaptic FM dye release was induced by applying 70 mM [K^+^] solution (Fig. 4b) or MCM (Fig. 4d) for 5 s through a microperfusion pipette positioned at a distance of approximately 100 μm from the respective neuron. Destaining of synaptic FM dye clusters, defined by a monoexponential loss of fluorescence, was in both cases observed. The amplitude and the time course of synaptic FM dye release have been shown to provide direct readouts of the vesicle pool size and the release probability, respectively, at the level of single synapses [34]. Thus, in order to analyze release amplitudes (*ΔF*) and time constants (*τ*), FM dye release traces of individual synapses were fitted by a mono-exponential function (Fig. 4e, f). MCM was able to induce an evident FM-unloading indicating MC mediators can induce synaptic vesicle release and retrieval in cultured neurons. However, depolarization with 70 mM [K^+^] solution induced a significantly higher (17.6 ± 1.4 a.u.; *p* = 0.002; *n* = 6 neurons) synaptic release amplitude than the MCM condition (8.7 ± 1.7 a.u; *n* = 14 neurons) (Fig. 4g), suggesting the cocktail of MC mediators results in a weaker stimulus and recruits a smaller pool of vesicles than the depolarizing stimulus. In contrast, the rate of release (time constants,*τ*) was similar (high K^+^, 12.06 ± 0.42 s; MCM, 11.44 ± 1.07 s) (Fig. 4h) indicating MCM has the same release efficacy of the individual synapses as depolarization with 70 mM [K^+^] solution.

### MC modulation of hippocampal neuronal function

**Fig. 5:**
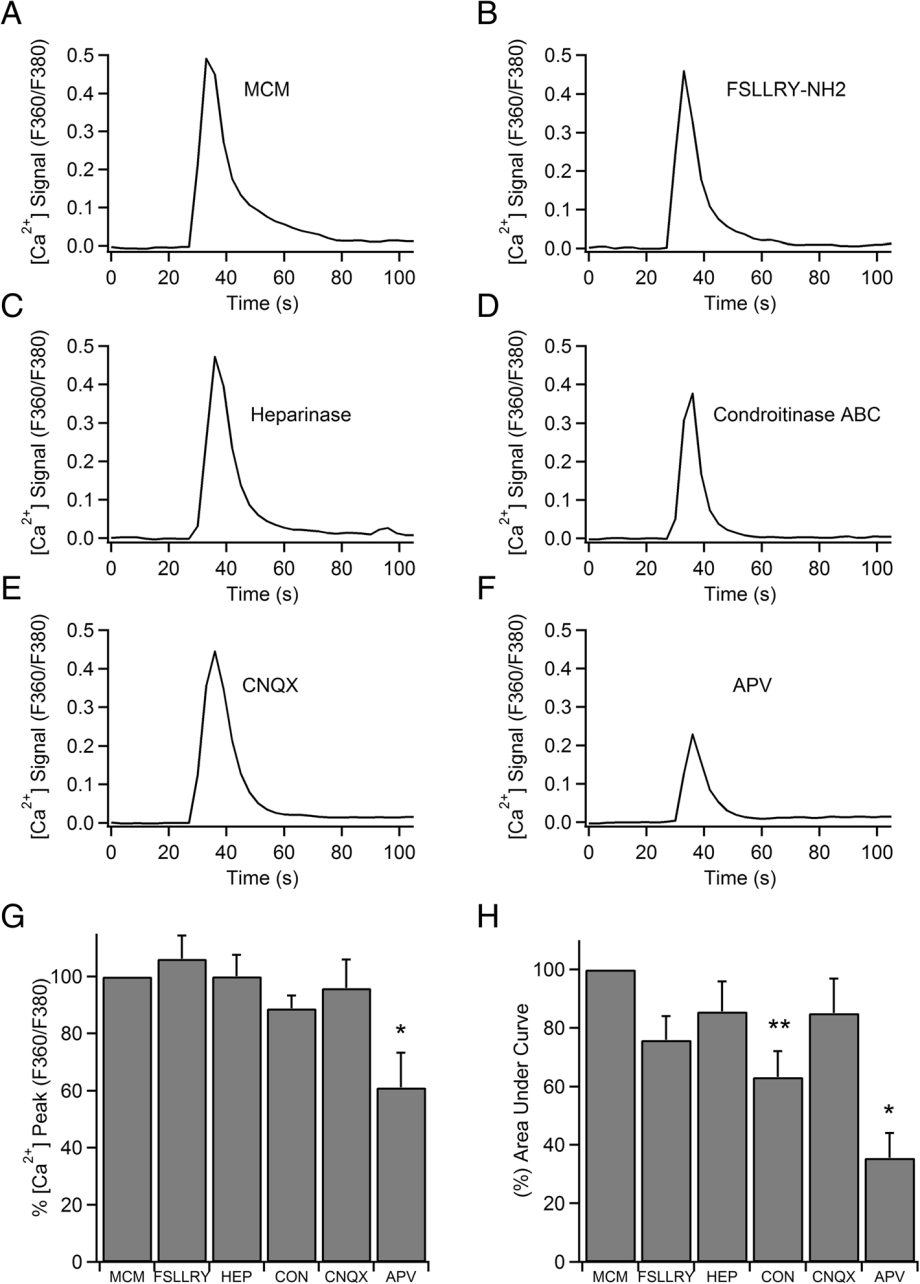
PGs of CS and NMDA-receptors contribute to outline the [Ca^2+^]_i_ signal elicited by mast cells mediator cocktail. **a** Representative time course of the elevation of [Ca^2+^]_i_ upon application of a brief pulse of MCM to a neuronal soma. **b** The response was not changed after incubation with FSLLRY-NH2 and **c** Heparinase, but **d** was decreased when MCM was pre-digested with enzyme chondroitinase ABC. **e**, **f** Examples of [Ca^2+^]_i_ signals elicited after CNQX 20 μM and D-APV 50 μM treatment. **g** Averaged results on the magnitude of the peak of [Ca^2+^]_i_ and **h** area under curve are presented as percentage of means ± SEM. Chondroitinase inhibited partially the [Ca^2+^]_i_ signal as well as the NMDA receptor selective antagonist, D-APV, that blocked [Ca^2+^]_i_ response by 65%; however, the AMPA and kainate receptors blocker, CNQX, did not affect it. *p < 0.05; **p < 0.01

Next, we investigated which mediators were involved in the MC-neuron signaling. MCs contain an array of chemical mediators that can be released to the extracellular medium upon activation. We focused on the preformed products contained in the granules since the brief delay in neuronal reaction after MC activation (≈ 30 s) suggests a rapid release of mediators by exocytosis. To elucidate which mediators were involved in the promotion of neuronal activation, we first directly stimulated neurons with bioactive monoamines (histamine, serotonin) and ATP. All of them failed to evoke a [Ca^2+^]_i_ transient in hippocampal neurons. Neuronal [Ca^2+^]_i_ signal remained at baseline even for long applications (data not shown), suggesting these mediators by themselves are incapable of eliciting the rapid response observed by MCM. Second, we selectively blocked PAR2 receptors with the antagonist FSLLRY-NH2 to determine the involvement of mast cell-specific proteases. We incubated cells for at least 10 min with FSLLRY-NH2 (400 μM) before proceeding to apply MCM. However, the drug barely modified [Ca^2+^]_i_ signal evoked by MCM (*ΔF*, 106.19 ± 8.13%; area under curve, 75.87 ± 8.16%; *n* = 66)(Fig. 5b). Lastly, to resolve the implication of highly anionic serglycin proteoglycans (PGs) containing glycosaminoglycan side chains of either heparin or chondroitin sulfate (CS) type in neuronal activation, we incubated the MCM with heparinase or chondroitinase ABC for 1 h at 37 °C to break down the PGs of heparin and CS, respectively. Heparinase did not change the peak amplitude and area under curve of [Ca^2+^]_i_ transients respect to untreated MCM (*ΔF*, 100.09 ± 7.7%; area under the curve, 85.6 ± 10.3%; *n* = 57) (Fig. 5c). Conversely, chondroitinase ABC reduced the amount of [Ca^2+^]_i_ entry into the cell (Fig. 5d). Although [Ca^2+^]_i_ amplitude did not decrease significantly (*ΔF*, 88.81 ± 4.47%), [Ca^2+^]_i_ transient was narrower than that observed by untreated MCM, resulting in a lower area under the curve (63.3 ± 8.89%; *p* = 0.006; *n* = 57) (Fig. 5h), suggesting PGs may be in part responsible of MC-neuron communication. Although, we combined both enzymes and increased incubation time of MCM to 24 h at 37 °C to make sure the complete degradation of PGs, we did not observe a stronger effect on [Ca^2+^]_i_ signal (data not shown). On the other hand, we decided to look into the mechanism that allows [Ca^2+^]_i_ to rise in neurons following MCM application. Accordingly, we examined the participation of glutamate receptors by using the NMDAR antagonist, D-2-amino 5-phosphonovaleric acid (D-APV), and the AMPA and kainate receptor blocker, 6-cyano-7-nitroquinoxaline-2,3-dione (CNQX). Cell incubation of D-APV 50 μM for 10 min at 37 °C before MCM perfusion achieved the most important reduction in [Ca^2+^]_i_ rise elicited by the cocktail of MC mediators (Fig. 5f) (*ΔF*, 61.15 ± 12.14%; *p* = 0.047; area under the curve, 35.54 ± 8.51%; *p* = 0.021; *n* = 25). However, the inhibition of AMPA and kainate receptors with CNQX 20 μM (after cell incubation for 10 min, at 37 °C) did not modify the [Ca^2+^]_i_ transient evoked by MCM (*ΔF*, 96 ± 9.9%; area under the curve, 85.04 ± 11.7%; *n* = 28) (Fig. 5e). In sum, the results of pharmacological experiments indicate that MC granule mediators elicit calcium transients and exocytosis by activating NMDA receptors in hippocampal neurons and partly, at least, PGs of CS rather than heparin contribute to the mediation of these effects on neuronal physiology.

## Discussion

The results presented here constitute the first report of bidirectional communication between MCs and hippocampal neurons at cellular level. We used [Ca^2+^]_i_ imaging of hippocampal neurons and peritoneal MCs in a coculture model to record their activation after a selective stimulation of both cells. MCs were activated when neurons were stimulated by depolarization through a pulse of 70 mM [K^+^] solution (Fig. 1). MC activation always occurred after neuronal response with a latency of 33 s which largely can reflect the diffusion time from neurotransmitters to MC target. In the present study, around 50% of all MCs in the field of view surrounding the stimulated neuron reply by means of a period of [Ca^2+^]_i_ oscillations. The failure to activate all MCs may be due to non-viable MCs, because of a lack of the necessary growth factors in the coculture that are required for mast cell survival (SCF, IL·3) [35]. Moreover, distinct populations of secretory vesicles containing diverse neuropeptides and transmitters have been reported in different types of inhibitory hippocampal neurons [36], and it is probable that MCs cannot respond to all of them. Interestingly, after MC responses, neurons were again able to evoke small [Ca^2+^]_i_ transients, suggesting MCs are able to release some of their mediators that feedback on neuronal activity. The short latency measured between MC response and the late onset [Ca^2+^]_i_ transient in neurons (≈ 30 s) suggests that involved mediators are preformed bioactive molecules released by exocytosis, because new synthesized lipid mediators and cytokines are generally released in the hours following mast cell activation [24]. MCs did not show an apparent change in morphology, which is indicative of full degranulation, rather reversible fusion or “kiss-and-run”: a mechanism of exocytosis involving the formation of a narrow fusion pore connecting transiently granule and plasma membranes that result in a slow and partial release of granule contents [37–39]. In MCs, the “kiss-and-run” mode would allow the secretion of small molecules contained in the granule, such as amines, but the complete release of large material such as PGs by full fusion would require stronger activation and degranulation [40]. Moreover, a mode of transient fusion referred to as cavicapture, in which fusion pore dilation allows the loss of cargo while the omega shape of the fusing granule is preserved, has also been described in MCs [27] and could also explain our observations. Therefore, a minor stimulation of brain MCs without degranulation may be sufficient to activate hippocampal neurons. Anyway, this argument is not in accordance with the observed degranulation of MCs stimulated by antigen (DNP) and C48/80, an apparent requirement to evoke neuronal activation (Fig. 2). LPS did not evoke MC degranulation nor neuronal response.

The cocktail of MC mediators obtained by heat for 1 h probably contains the whole arsenal of inflammatory mediators and biologically active substances. A pulse of this MCM elicited a consistent and reproducible [Ca^2+^]_i_ signaling in hippocampal neurons (Fig. 3). When diluted, the cocktail was able to evoke a neuronal signaling up to a 1:10 dilution, which contains the mediators extracted from around 100,000 MCs per milliliter. Brain MCs are not numerous [5, 41]. However, their number and distribution can dramatically change in response to a number of environmental stimuli such as trauma and stress [42, 43]. In addition, the number of infiltrated MCs increases in patients with Alzheimer’s disease compared to healthy subjects [44]. Likely, both amounts of activated MCs and level of MC activation may determine the type and amount of released inflammatory mediators and influence brain state.

Ca^2+^ influx mediates synaptic release, which is physiologically triggered by trains of action potentials [45]. Similar to bursts of action potentials, high [K^+^] treatment results in sustained elevations of the presynaptic Ca^2+^ concentration. Ca^2+^ transients result in FM dye destaining which represents the balance of vesicle fusion and vesicle endocytosis [46, 47]. We used quantification of FM dye release induced by the cocktail of MC mediators in cultured hippocampal neurons to determine their contribution to transmitter release. By analyzing the magnitude of release (*ΔF*) in individual synapses we demonstrate that the MCM yielded synaptic release associated with FM dye destaining, which can be discriminated from the depolarization by 70 mM KCl (Fig. 4). The loss of FM fluorescence induced by MCM stimulation was 50% lower than that observed by depolarization. This difference is expected because [Ca^2+^]_i_ transients evoked by MCM also showed lower peaks than those evoked by depolarization.

Neuronal regulation of MCs involves classical neurotransmitters such as acetylcholine, neuropeptides such as substance P, CGRP, vasoactive intestinal peptide (VIP), and neurotensin [48]. For instance, there has already been reported bidirectional signaling between MCs and submucous neurons in the human gut and that both VIP and CGRP are the neuropeptides involved in intestinal MC activation [49]. In the hippocampus, neuropeptide Y is present in a subpopulation of nonpyramidal cells [50] and it might be responsible for MC activation by hippocampal neurons in our coculture model. It will be necessary to evaluate this hypothesis in the future. On the other hand, pharmacological dissection of MC mediators to discriminate those able to activate hippocampal neurons is quite complex. Despite that we select only preformed mediators to evaluate the involvement in neuronal activation, there are too many alternatives because MCs have the unique ability to store and release certain preformed cytokines and growth factors, including tumor necrosis factor (TNF) [51] and vascular endothelial growth factor [52]. Actually, TNF can be rapidly released (first 10 min from MC granules) and subsequently secreted along with other pro-inflammatory cytokines with a new synthesis after several hours [53]. If release of involved mediators occurs by “kiss-and-run”, PGs are unlikely to evoke neuronal activation because the high molecular weight of PGs [54] demands the complete fusion of granules (as explained above). However, solely pre-digestion with chondroitinase ABC produced a significant reduction in [Ca^2+^]_i_ transients evoked by the MCM (Fig. 5). Peritoneal MCs contain both heparin and galactosaminoglycan dermatan sulfate (chondroitin sulfate B) in their granules as demonstrated by analysis of the GAGs by using nuclear magnetic resonance spectroscopy [55]. It was reported that chondroitin sulfate is able to elicit inward currents, cell depolarization, and calcium transients by acting on AMPA and kainate receptors of hippocampal neurons [56, 57]; therefore, it is not surprising we found that pretreated MCM with chondroitinase ABC was able to reduce [Ca^2+^]_i_ transients in hippocampal neurons. However, in our experiments, NMDA receptors as opposed to AMPA and kainate receptors seem to mediate neuronal activation by MCM. Without considering new synthesized mediators, granules contain a cocktail of preformed mediators that can be rapidly released. Although, one by one, neither histamine, serotonin, nor ATP were able to elicit a [Ca^2+^]_i_ transient in neurons, the combined action of substances from activated MCs may be different from those of single agents and complicated interactions may occur between MC mediators and the potential neuronal targets. Indeed, histamine by itself did not evoke an observable rise in [Ca^2+^]_i_ signal. However, it was reported that histamine potentiates NMDA-mediated synaptic transmission and excitotoxicity in hippocampal neurons [58, 59]. When histamine is co-released simultaneously with CS, as it should occur during full exocytosis, the effects on synaptic transmission in the hippocampus must be more robust and even toxic than when histamine is released solely by the more subtle mechanism of “kiss-and-run”. Thereby, the combined effects of MC mediators can have unsuspected consequences on neuronal characteristics. It may be particularly decisive for the inflammatory state of the cerebral tissue the contribution of MC cytokines. MCs can be activated by various molecules through the toll-like, interleukin-1, and neurotransmitter receptors and secrete pro-inflammatory cytokines. TNF and interleukin-1 family cytokines play a crucial role in the pathogenesis and management of the CNS inflammatory response [53]. Interestingly, it has been proposed the use of IL-37 (a cytokine member of the IL-1 family with immunosuppressive and anti-inflammatory properties) in neuroinflammation to help to improve the course of many neurological diseases [60, 61].

## Conclusions

Here, we demonstrated bidirectional communication between MCs and hippocampal neurons with important effects on Ca^2+^ influx and neurotransmission. Increasingly, new evidence points towards MCs in neuroinflammation and brain disorders such as cerebral ischemia, multiple sclerosis, Alzheimer’s disease, or depression [62]. As MC is a long-lived innate immune cell that responds to its microenvironment through a range of surface receptors and even to toxic molecules such as amyloid beta peptides [63], the accumulation of toxics in the brain may create a positive feedback loop and a sustained activation of MCs that can result in neuronal [Ca^2+^]_i_ overload and long-term neuronal damage.

## Abbreviations

[Ca^2+^]_i_: Intracellular calcium concentration
CNQX: 6-Cyano-7-nitroquinoxaline-2,3-dione
CNS: Central Nervous System
CS: Chondroitin sulfate
D-APV: D-2-amino 5-phosphonovaleric acid
DNP: Dinitrophenyl
LPS: Lipopolysaccharide
MC: Mast cell
MCM: Cocktail of MC mediators
NMDAR: N-methyl-D-aspartate receptor
PG: Proteoglycans
TNF: Tumor necrosis factor
